# Modeling of the combined dynamics of leptospirosis transmission and seroconversion in herds

**DOI:** 10.1038/s41598-022-19833-x

**Published:** 2022-09-16

**Authors:** Sudarat Chadsuthi, Karine Chalvet-Monfray, Angeli Kodjo, Anuwat Wiratsudakul, Dominique J. Bicout

**Affiliations:** 1 Department of Physics, Research Center for Academic Excellence in Applied Physics, Faculty of Science, Naresuan University, Phitsanulok, 65000 Thailand; 2 Université de Lyon, INRAE, VetAgro Sup, UMR EPIA, 69280 Marcy l’Etoile, France; 3 Université Clermont Auvergne, INRAE, VetAgro Sup, UMR EPIA, 63122 Saint Genès Champanelle, France; 4 USC 1233, Laboratoire des Leptospires, VetAgro Sup, 69280 Marcy l’Etoile, France; 5 Department of Clinical Sciences and Public Health, and the Monitoring and Surveillance Center for Zoonotic Diseases in Wildlife and Exotic Animals, Faculty of Veterinary Science, Mahidol University, Nakhon Pathom, 73170 Thailand; 6 Univ. Grenoble Alpes, CNRS, Grenoble INP, VetAgro Sup, TIMC, 38000 Grenoble, France; 7 Laue-Langevin Institute, Theory Group, 71 Avenue des Martyrs, 38042 Grenoble, France

**Keywords:** Diseases, Infectious diseases

## Abstract

Leptospirosis is a zoonotic disease-causing illness in both humans and animals resulting in related economic impacts due to production loss as well as prevention and control efforts. Several mathematical models have been proposed to study the dynamics of infection but none of them has so far taken into account the dynamics of seroconversion. In this study, we have developed a general framework, based on the kinetic model for animal leptospirosis, that combines both the antibody (exposure marker) and infection dynamics to simultaneously follows both seroconversion and infection status of leptospirosis in a herd population. It is a stochastic compartmental model (for transition rates) with time delay (for seroconversion) which describes the progression of infection by a SEIRS (susceptible, exposed, infected, removed and susceptible) approach and seroconversion by four-state antibody kinetics (antibody negative and three antibody positive states of different antibody levels). The model shows that it is possible to assess and follow both seroconversion and infection status through the prism of diagnostic testing. Such an approach of combined kinetics could prove very useful to assist the competent authorities in their analyzes of epidemic situations and in the implementation of strategies for controlling and managing the associated risks.

## Introduction

Leptospirosis is caused by pathogenic spirochetes belonging to the genus *Leptospira* of the family Leptospiraceae^1^. It is a zoonotic disease found in human and animals worldwide. However, the disease is prevalent mainly in subtropical or tropical countries where humid climates abide^2^. Humans and animals are infected after encountering either the waste products of infected animals or exposed to an environment that is contaminated with leptospires. Leptospires can enter through the body via small cuts, mucous membranes, or through wet skin^3^.

Leptospires can be present in animals especially rodents, small marsupials, dogs, and livestock. A susceptible animal can become infected with Leptospira through direct or indirect contact with infected urine or tissues of infected animals. In animals, the leptospirosis associated symptoms vary with infected serovars or host adapted serovars^3,4^. Some of the infected animals remain asymptomatic and may shed the bacteria in their urine for life^5^. The carriage of leptospires in the proximal renal tubules leads to contamination of soil, water, and rivers. This becomes the main sources of transmission. Leptospirosis in animals is a disease with a major worldwide economic impact. Clinical signs of the leptospirosis infection in cattle are mainly manifested by reproductive problems such as infertility, abortion and weak offspring^6^.

Due to a varied and unspecific symptomatology, diagnosis tests depend on the laboratory assays used to identify leptospirosis infection. Laboratory techniques such as the detection of specific antibodies, microscopic agglutination test (MAT), indirect hemagglutination assay (IHA), and immuno-enzymatic assays (ELISA) are keys to detect the carriage of *Leptospira*. MAT is the most common and standard technique used in serological examination as it produces high sensitivity and specificity results^7^. In addition, MAT is used to identify the circulated *Leptospira* serogroups^8–10^ by simultaneously detecting both immunoglobulin M (IgM) and immunoglobulin G (IgG), which are classes of agglutinating antibodies^11,12^. Nonetheless, ELISA can differentiate IgM or IgG antibodies^10,13^. To detect the antigen, PCR is an alternative. This method can directly detect leptospiral DNA in the samples. However, it cannot identify the etiological serovars^12^. The isolation and identification of *Leptospira* is possible with the culture method. Nevertheless, the method is difficult for many reasons, for examples, type of samples and the timing of taken samples^12^. Therefore, all currently available leptospirosis infection diagnostic tests could not provide a definite indication.

To study the infection dynamics of leptospirosis, several mathematical models have been proposed. A Susceptible—Infectious (SI) epidemiological model was used for the spread of leptospirosis in livestock by varying periodic parameters^14^ and for the leptospire dynamics and control in the Norway rat^15^. A Susceptible—Infectious—Retired (SIR) model was used to study the transmission of leptospirosis between human and animal populations^16,17^. This SIR model was then used to understand the epidemiology of leptospirosis in cattle^18^. To improve the description of the dynamics of infection with *Leptospira*, a SEIR (SIR plus the “Exposed” class) model was used to study the optimal control of disease outbreak^19^. However, those models mainly focused on the sensitivity of the transmission parameters that are related to the number of infected animals based on the simulation results. Nevertheless, the time series of infected animals are not well defined due to the limitations of current diagnosis methods. To follow the spread of the disease in the animal population, serological diagnostics are widely used and accepted. However, to our knowledge, the seroconversion kinetics of leptospirosis have never been taken into account in previous mathematical models cited above. Indeed, by supplementing those models on the dynamics of the infection by tracking the level of antibodies (exposure marker or probe) makes it possible to have a more detailed view of the state of the circulation of pathogens in the considered population and to improve the interpretations of diagnostic tests.

In this study, we aim to assess the epidemiological correlations between the seroconversion dynamics, as obtained from serological diagnostic tests, and infectious status of a population of animals during an outbreak. To this end, we built a general framework based on the kinetic model for animal leptospirosis that combines both the infection and the antibody dynamics. Using a compartmental model with time delay, the model simultaneously follows both infection and seroconversion dynamics. The states of the infection and antibody classes of the population are described as a function of the basic reproduction number allowing the correlation between the number of infections (epizootic size) and the prevalence of antibody-positive individuals.

## Results

### Seroconversion dynamical model

**Figure 1:**
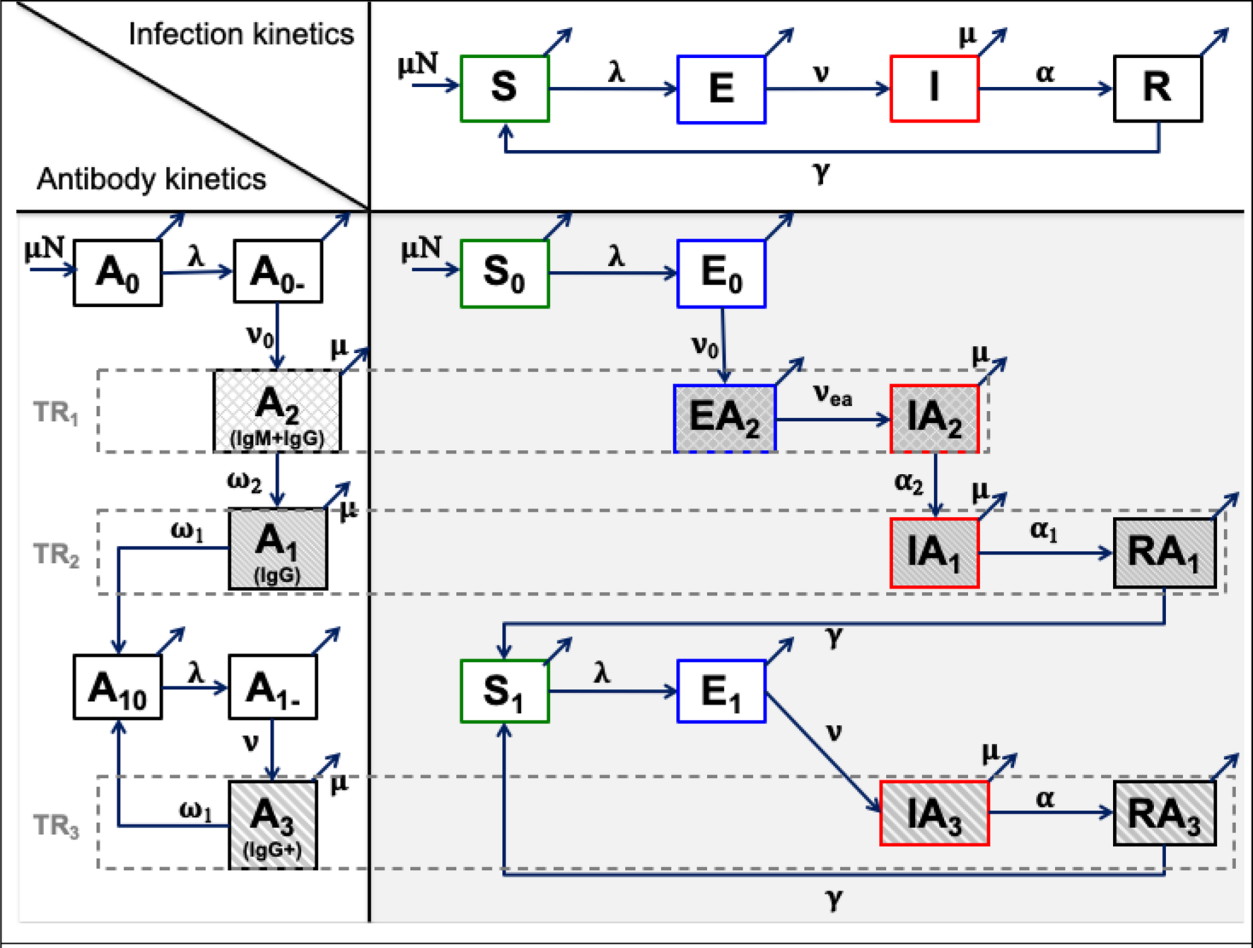
Kinetic scheme of the combined (right bottom) infection (top) and antibody (left bottom) dynamics. Description of population and parameters are provided in the main text and Table 1. $TR_{i}$ stands for “Test Result” of a diagnostic test. Arrows tilted up correspond to the natural mortality rate μ (identical for all arrows but just indicated for some).

A first key result of this study is the description of the general framework of the seroconversion dynamical model consisted of a combination of the infection and antibody dynamics. The kinetic schemes of the infection dynamics, antibody dynamics and combined model are shown in Fig. 1 and the equations are described in the “Methods” section.

**Table 1:** Parameters and time durations of the infection and antibody dynamics.

Definitions	Symbol	Mean [min, max]	Unit	References
Transmission rate	$\beta$	Varying	1/day	From Eq. (5)
Duration of incubation	$t_{e} = 1/\nu$	10	day	Baker, 1948^27^
Duration of infection	$t_{i} = 1/\alpha$	240 [200, 280]	day	Leonard, 1993^28^
Duration of immunity	$t_{r} = 1/\gamma$	542 [360, 720]	day	Estimated
Natural mortality rate	$\mu$	6.85 × 10^–4^	1/day	Estimated
Onset of IgM and IgG: primary infection	$1/\nu_{0}$	7	day	Cousins, 1985; Smith, 1994^20,29^
Onset of IgG+: subsequent infection	$1/\nu$	10	day	Estimated
Duration of IgM	$1/\omega_{2}$	65 [60, 70]	day	Leonard, 1993^28^
Duration of IgG	$1/\omega_{1}$	782	day	Estimated
Duration of incubation (EA_2_ → IA_2_)	$t_{ea} = 1/\nu_{ea}$	3 [1, 5]	day	Estimated
Duration of seroconversion from IgM to IgG (IA_2_ → IA_1_)	$t_{ia2} = 1/\alpha_{2}$	$1/\omega_{2} - t_{ea}$	day	Calculated
Duration of infection (IA_1_ → RA_1_)	$t_{ia1} = 1/\alpha_{1}$	$t_{i} - t_{ia2}$	day	Calculated
Duration of immunity and IgG (RA_1_)	$t_{ra1}$	$t_{r}$	day	Calculated
Duration of infection for IA_3_ state	$t_{ia3}$	$t_{i}$	day	Calculated
Duration of immunity and IgG+ (RA_3_)	$t_{ra3}$	$t_{r}$	day	Calculated
Basic reproduction number	$R_{0}$	Varying	–	Equation (4)

The infection dynamics was explained following the compartmental Susceptible-Exposed-Infectious-Recovered-Susceptible (SEIRS) model. Rather than a SIR model like in some works cited above, we have chosen to use a SEIRS model to allow a stage (compartment E) infected but not yet infectious and which may or may not already be carrying antibodies. Indeed, the development and appearance of antibodies taking place during the incubation phase, such a stage is quite plausible and possible. The SEIRS model is richer and can be reduced to a SIRS model when the duration of stage E turns out to be very short; the reverse not being possible. Moreover, as we used a stochastic SEIRS model with residence times in the stages obtained from the constrained distributions of Eq. (6), epidemiological situations with or without stage E are statistically possible.

Infectious animals can transmit the disease to susceptible animals that progress to exposed animals with a transmission rate $\lambda$. Exposed animals became infectious after an incubation period of mean duration $t_{e} = 1/\nu$. The mean duration of infection is $t_{i} = 1/\alpha$. Infectious animals progress to recovered and immune state, which became susceptible again after a mean duration of immunity of $t_{r} = 1/\gamma$. All animals die at a mortality rate $\mu$ considered as the population renewal rate. The set of equations related to the dynamics of infection were provided in the Supplement Information (S1. Methods).

Regarding the antibody kinetics, the model is based on the antibody level of animals (mainly cattle here). Primarily exposed animals developed antibody levels from negative to positive in both IgM and IgG antibodies and later only positive IgG antibodies. IgM antibodies stay for 3–5 weeks (in cattle)^20^ while IgG antibodies, appearing at the same time or just after IgM antibodies, stay for a much longer time^20^. Subsequently, the animal IgG antibodies decrease at a rate $\omega_{1}$ and became negative again in both IgM and IgG antibodies. Afterwards, re-infection of those animals rose their IgG antibodies to a higher level^21,22^ (IgG + state).

The infection and the antibody kinetics are combined to study the seroconversion dynamics. When susceptible animals were first infected with leptospires, they enter exposed state with negative antibodies. Subsequently, exposed animals develop IgM and IgG antibodies at a rate $\nu_{0}$, and then progress to the infectious state at a rate $\nu_{ea}$. However, infectious animals that recovered still carried IgG antibodies during the immunity period and became susceptible again with negative results in both IgM and IgG antibodies at a rate $\gamma$.

### Model outcomes

**Figure 2:**
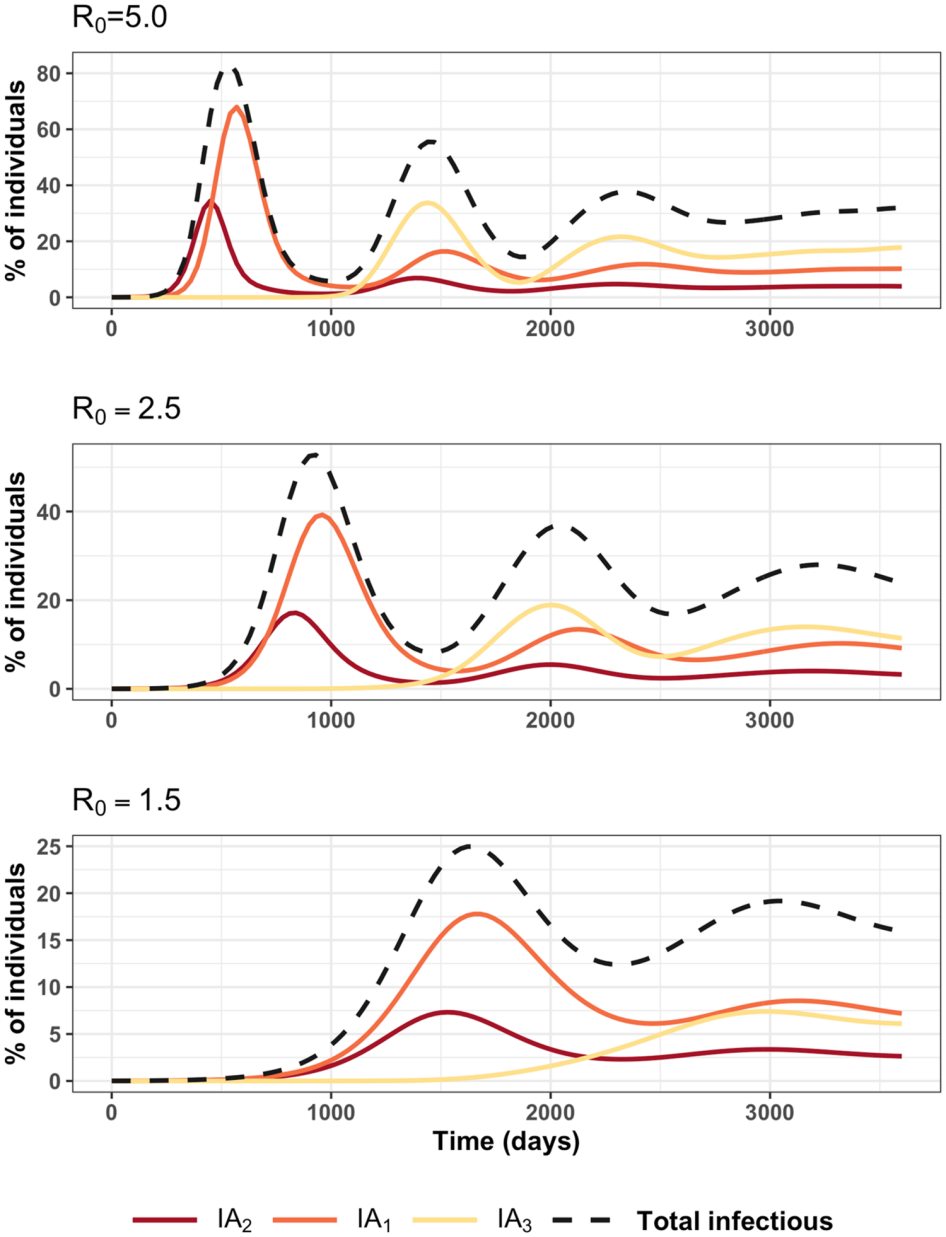
Average proportion of infected animals of type “$i$” ($IA_{2}$: carrying IgM and IgG antibodies, $IA_{1}$: only carrying IgG and $IA_{3}$ carrying high-level of IgG) as a function of time for various values of $R_{0}$. Proportion = 100 × number of animals / total population; with total population = 10,000.

The average number of infectious animals (i.e., $IA_{2}$, $IA_{1}$ and $IA_{3}$ compartments) are shown in Fig. 2 for the basic reproduction number $R_{0}$ equal to 1.5, 2.5, and 5.0. All results of the 10 compartments are shown in Fig. S1. As a check, the comparisons between the combined model and the reduced SEIRS model are shown in Fig. S2. At the beginning, there was no circulation of leptospirosis infection, the epidemic curve begins to increase and then oscillates around a plateau. The results showed that the epidemic curve could be divided into two phases: a growth phase and a stationary state. During the growth phase, the total infectious animals is mainly composed of $IA_{2}$ and $IA_{1}$. After that, the total infected population consists of the number of $IA_{3}$, $IA_{2}$, and $IA_{1}$, where most of the infectious animals came from $IA_{3}$.

For $R_{0} > 1$, the first peak of infectious animals comes from the number of $IA_{2}$, carrying IgM and IgG antibodies, followed by $IA_{1}$ and $IA_{3}$ carrying IgG and high-level of IgG antibodies, respectively. The time interval between first peaks of each infectious compartments changed with $R_{0}$. For all value of $R_{0}$, the time interval between peaks of $IA_{2}$–$IA_{1}$ is about twice of the IgM duration ($1/\omega_{2}$). The time interval between peaks of $IA_{2}$–$IA_{3}$ and $IA_{1}$–$IA_{3}$ decreased as $R_{0}$ increases. Increasing IgM duration leads to increase the time interval between peaks of $IA_{2}$–$IA_{3}$, and $IA_{1}$–$IA_{3}$, but does not affect the time interval between peaks of $IA_{2}$–$IA_{1}$ (data not shown). The cross-correlations of the monthly number of $IA_{2}$, $IA_{1}$ and $IA_{3}$ are plotted in Fig. S3. A positive strong correlation between $IA_{1}$ and $IA_{2}$ with a positive 4-month lag was found for all value of $R_{0}$, excepted for $R_{0}$ = 1.5, the lag time was 3 months. This 4-month lag corresponds to the time interval between the peaks of the number of $IA_{2}$ and $IA_{1}$ (Fig. 2). Overall, the correlation between $IA_{3}$–$IA_{2}$ and $IA_{3}$–$IA_{1}$ were similar excepted for $R_{0} = 1.5$. From a − 1.5 to 1.5 years lag, the negative correlations between $IA_{3}$–$IA_{2}$ and $IA_{3}$–$IA_{1}$ were found, which implied that the high number of $IA_{2}$ and $IA_{1}$ related to the low number of $IA_{3}$. The simulation results can be used to forecast the number of $IA_{1}$ and $IA_{3}$ at later times from the number of $IA_{2}$ in the past (occurring first); that is to say, when the number of new infectious $IA_{2}$ was observed, the number of $IA_{1}$ or $IA_{3}$ can be predicted. Prior to the stationary state, Fig. 2 shows that there is no general relation in the numbers of infected animals as a function of $R_{0}$.

### Characteristics of infectious

**Figure 3:**
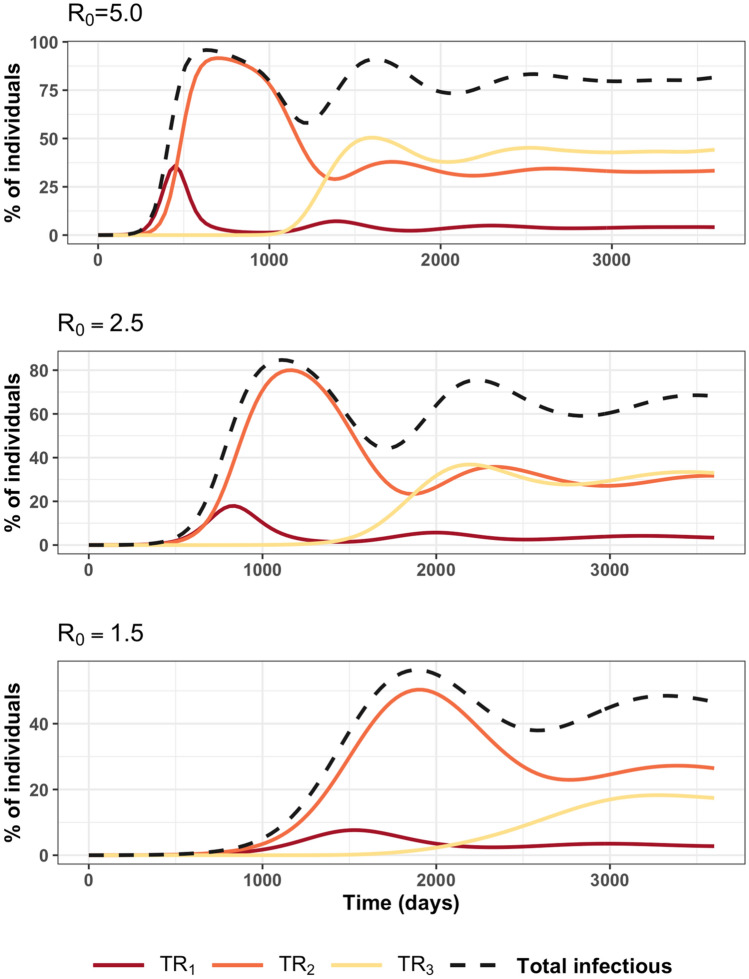
Average proportion of antibody positive animals of type “$i$” from the diagnostic test ($TR_{1}$, carrying IgM and IgG antibodies; $TR_{1}$, only carrying IgG and $TR_{3}$ carrying high-level of IgG) for 10 years. Proportion = 100 × number of animals/total population; with total population = 10,000.

**Figure 4:**
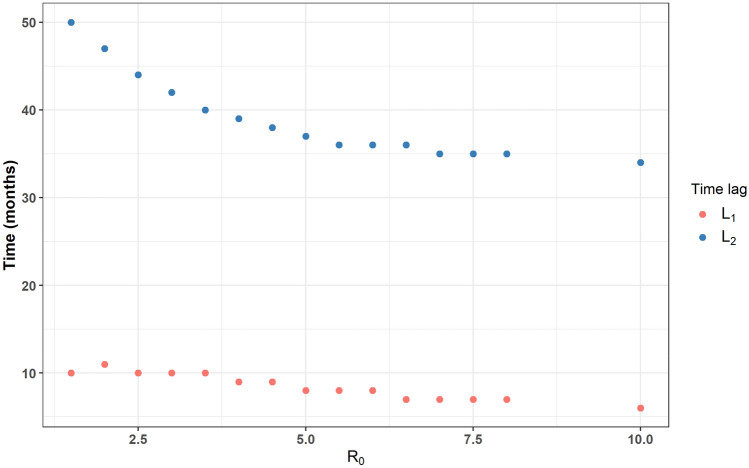
Lag times $L_{1}$ (between test results $TR_{2}$ and $TR_{1}$) and $L_{2}$ (between test results $TR_{3}$ and $TR_{1}$) as a function of $R_{0}$.

Diagnosis tests such as ELISA can be used to inform the public health about the situation concerning the number of infected in each animal herd. The number of infected animals is derived and classified as the numbers of $TR_{1}$, $TR_{2}$, and $TR_{3}$ (as shown in Fig. 6 in “Methods” section). The total number of infectious and that of $TR_{1}$, $TR_{2}$, and $TR_{3}$ are shown in Fig. 3. Both Figs. 2 and 3 exhibit similar trends as a function of time. During the growth phase, the total number of infectious animals is mainly composed of the number of $TR_{1}$ and $TR_{2}$, afterward it tends to be mainly $TR_{2}$ and $TR_{3}$. The cross-correlations between the number of $TR_{i}$ are shown in Fig. S4. Overall, the patterns of the cross-correlations between the number of infectious animals were similar to the number of $TR_{i}$. The positive strong correlations between $TR_{1}$ and $TR_{2}$ were found to decrease as $R_{0}$ increase. This indicates that the number of $TR_{2}$ can be forecasted from the number of $TR_{1}$ with a lag time $L_{1}$. Figure 4 shows that $L_{1}$ tends to decrease a bit with $R_{0}$. However, positive weak correlations between the number of $TR_{3}$ and $TR_{1}$ were observed at lag times ($L_{2}$) of 50, 44, 37 and 35 months for $R_{0}$ = 1.5, 2.5, 5.0 and 7.5, respectively. As shown in Fig. 4, $L_{2}$ decreases with $R_{0}$.

The comparison of results between the number of infectious $IA_{i}$ and the number of $TR_{i}$ for $R_{0}$ = 1.5, 2.5, and 5.0 is shown in Fig. S5. The graph indicates that the number of $TR_{i}$ perfectly parallel the number of infectious animals (with, $TR_{1} \sim IA_{2}$) all the time both in the growth and stationary phases. Therefore, the number of $TR_{i} { }$ can be used to extract the proportion of infectious animals any time. To estimate a cut-off time between growth and stationary phases, the relaxation time (time to a stationary state or a cut-off time ($t_{c}$)) is defined as the time reached at the 1st minimum of $TR_{i}$ after the 1st peak of that curve. The average number of $TR_{i}$ at stationary state is calculated as the average number over the period since the relaxation time to the end of simulation time (30 years). Values of $t_{c}$ for $TR_{i}$ are provided in Table S2.

**Figure 5:**
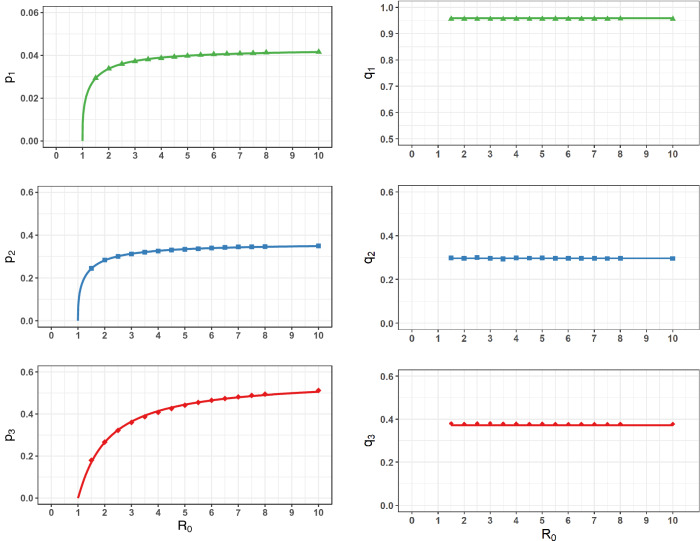
Fractions of antibody positive and infected animals from the diagnostic test results (1, carrying IgM and IgG antibodies; 2, only carrying IgG and 3, carrying high-level of IgG) at 10 years as a function of $R_{0}$. $p_{i}$ stands for the probability of antibody positive results of type “$i$” and $q_{i}$ for the probability of infected individuals among antibody positives of type “$i$”. Solid lines through the data are the best-fit to the data (symbols) with Eq. (9) for $p_{i}$ (left panel) and represent Eq. (10) for $q_{i}$ (right panel). Dashed lines representing the 95% credible intervals are not visible.

The average number of $TR_{i}$ at stationary state was used to calculate the probability $p_{i}$ of finding antibody positive animals in $TR_{i}$ as a function of $R_{0}$ (Fig. 5, left panel). Clearly, $p_{i}$ is equal to zero for $R_{0} \le 1$ and increases with $R_{0}$. However, the probability $q_{i}$ of finding infected individuals among the positive $TR_{i}$ showed no variation with $R_{0}$ (Fig. 5, right panel) as predicted from Eq. (10) for the stationary state expression of $q_{i,s}$.

## Discussion and conclusions

Leptospirosis is a zoonotic disease-causing illness in both humans and animals resulting in related economic impacts due to production loss as well as prevention and control efforts^23^. Several mathematical models have been constructed to study the dynamics of infection using compartment models, such as the SIR^16–18^ and SEIR models^19^. These models did not consider the seroconversion dynamics. In this study, a general framework that combines both antigen and antibody dynamics was constructed to assess the state of leptospirosis in a population of animals using diagnostic tests. To our knowledge, this model is the first compartment model built to access leptospirosis progression profile in animals considering the IgM and IgG antibody kinetics and outcomes of diagnosis tests.

It is well known that the clinical signs in animals are mild or asymptomatic^24^. Thus, the situation of leptospirosis outbreak is only described through the test results from sampling population. In our framework, a novel approach to explore the dynamics of leptospirosis in animals was proposed with the number of infected animals derived from the test results.

Using IgM-ELISA, for example, with sensitivity 86% ($Se_{1}$) and specificity 84% ($Sp_{1}$)^25^, the fraction of positive $T_{ + ,i}$ makes it possible to calculate the prevalence $p_{1}$ or the true positive antibody fraction of animals. This is helpful to uncover the actual epidemic situation rather than relying on only the serological test results. Furthermore, the basic reproduction number ($R_{0}$) is estimated using Eq. (9). In general, $R_{0}$ is a useful tool for describing and predicting the dynamics of infection. In mathematical models^15,26^, $R_{0}$ is defined from the transmission rate, a parameter that is difficult to access. In this study, $R_{0}$ can be prior calculated to infer the transmission rate. Subsequently, after the parameters in Eq. (10) are known, the fractions of infected states among the positive tests can be calculated. Thus, our model is useful to elucidate the picture of leptospirosis dynamics either from the parameters of Eq. (10) or from the data set from the field.

Furthermore, the cross-correlation of the number of $TR_{i}$ was studied to provide better understanding on the time lag of infection. Animals with positive antibody is anticipated in later time after the outbreak. Using the time lag, for example, if the number of $TR_{1}$ is estimated, the number of $TR_{2}$ and $TR_{3}$ can be then predicted. Reversely, if the high titer level of IgG is known by other means the number of $TR_{3}$ can be calculated and the $TR_{1}$ and $TR_{2}$ in the past can be tracked back. The time lag output is based on the input parameters and is flexible to change. Thus, this model is also applicable and can be adapted for leptospirosis in humans once data collected from human outbreaks and relevant diagnostic tests is available.

The developed model that we have just described is based on only on few assumptions and the outcomes depend on the quality of the parameters used. The model considered a closed system with a homogeneously well-mixed population with the parameters used obtained from the literature (see Table 1). To take account for the uncertainties in the parameters we used distributions to sample the parameters. It was assumed that the parameters were constant over the entire study period. The model can be embellished in several directions including age structure and precise renewal pattern of populations, spatio-temporal aspects or other factors that may have an impact on the transmission of leptospires like weather conditions.

In conclusion, we developed and constructed a general and generic modeling framework allowing to describe the transmission dynamics of animal leptospirosis in which antibody kinetics is taken into account. The model also makes it possible to describe the outbreak situations through the prism of diagnostic tests. This proposed new approach could prove useful to the competent authorities in their analyzes of real outbreak situations and, consequently, in the implementation of disease control and associated risk management strategies.

## Methods

### Seroconversion dynamical model

The kinetics of antibody levels in an animal with leptospirosis can be described as follows (Fig. 1, left bottom). $A_{0}$ represents the numbers of naïve animals that have never encountered leptospires and therefore do not carry associated antibodies. The exposure of $A_{0}$ to leptospires results to animals infected for the first time and still antibody-negative, $A_{0 - }$, which evolves after a while towards $A_{2}$ carrying both IgM and IgG antibodies. Next, $A_{2}$ loses IgM antibodies and progresses to $A_{1}$ carrying only IgG antibody. When $A_{1}$ loses its antibodies, it becomes $A_{10}$ again susceptible to secondary and subsequent leptospiral infections. On the other hand, $A_{1 - }$ resulting from an infection of $A_{1}$, evolves towards an $A_{3}$ state carrying only IgG but at a high level compared to $A_{2}$ and $A_{1}$. And $A_{3}$ becomes, $A_{10}$, susceptible again to infections when it loses its antibodies.

Now, to build the overall model, this antibody kinetics was combined with the infection dynamics according to a SEIRS model as follows. Let $N$ be the constant total number of individuals or animals in the population. The total population is subdivided into ten compartments where (chronologically according to Fig. 1, right bottom):

- $S_{0}$: number of susceptible animals that have never been neither exposed to nor infected with leptospires. $S_{0}$ are antibodies negative.
- $E_{0}$: number of exposed animals that have been infected with leptospires for the first time. It is the $S_{0}$ that becomes $E_{0}$ after exposure and infection with pathogens. $E_{0}$ are infected but not infectious yet. $E_{0}$ are antibodies negative.
- $EA_{2}$: number of animals exposed and infected (not infectious) for the first time and carrying both IgM and IgG antibodies. $EA_{2}$ follows after $E_{0}$.
- $IA_{2}$: number of animals infected for the first time and now infectious (capable of transmission to susceptible animals) and carrying both IgM and IgG antibodies. $IA_{2}$ follows after $EA_{2}$.
- $IA_{1}$: number of animals infected for the first time and still infectious but carrying only IgG antibodies. $IA_{1}$ follows after $IA_{2}$ loses IgM antibodies.
- $RA_{1}$: number of animals infected for the first time that have recovered (no longer infectious) and still carrying IgG antibodies. $RA_{1}$ follows after $IA_{1}$ loses the infection following recovery.
- $S_{1}$: number of animals susceptible to leptospiral infection but having already had a history of infection and not carrying associated antibodies. $S_{1}$ follows after $RA_{1}$ or $RA_{3}$ loses the antibodies.
- $E_{1}$: number of exposed animals having already had a history of infection and not carrying associated antibodies. It is the $S_{1}$ that becomes $E_{1}$ after secondary and subsequent infections with pathogens. $E_{1}$ are infected but not infectious yet.
- $IA_{3}$: number of infected animals (secondary and subsequent infections) that are infectious (capable of transmission to susceptible animals) and carrying high level (greater than in both $IA_{2}$ and $IA_{1}$) IgG antibodies. $IA_{3}$ follows after $E_{1}$.
- $RA_{3}$: number of infected animals (secondary and subsequent infections) that have recovered (no longer infectious) and still carrying high level IgG antibodies. $RA_{3}$ follows after $IA_{3}$ loses the infection following recovery.

And, let $\lambda \left( t \right) = \left( {\beta /N} \right) \times \left[ {IA_{2} \left( t \right) + IA_{1} \left( t \right) + IA_{3} \left( t \right)} \right]$ represents the time-dependent force of infection. The combined kinetics of infection and antibodies dynamics, according to Fig. 1, can be described by the set of ten delayed differential equations as:

1 $$\left\{ {\begin{array}{*{20}l} {\frac{{dS_{0} ~}}{{dt}} = \mu N - \left[ {\lambda \left( t \right) + \mu } \right]S_{0} } \hfill \\ {\frac{{dE_{0} ~}}{{dt}} = \lambda \left( t \right)S_{0} - \left[ {\nu _{0} + \mu } \right]E_{0} } \hfill \\ {\frac{{dEA_{2} ~}}{{dt}} = \nu _{0} \left[ {E_{0} \left( t \right) - \phi \left( {t_{{ea}} } \right)E_{0} \left( {t - t_{{ea}} } \right)} \right] - \mu EA_{2} } \hfill \\ {\frac{{dIA_{2} ~}}{{dt}} = \nu _{0} \phi \left( {t_{{ea}} } \right)\left[ {E_{0} \left( {t - t_{{ea}} } \right) - \phi \left( {t_{{ia2}} } \right)E_{0} \left( {t - t_{{ea}} - t_{{ia2}} } \right)} \right] - \mu IA_{2} } \hfill \\ {\frac{{dIA_{1} ~}}{{dt}} = \nu _{0} \phi \left( {t_{{ea}} } \right)\phi \left( {t_{{ia2}} } \right)\left[ {E_{0} \left( {t - t_{{ea}} - t_{{ia2}} } \right) - \phi \left( {t_{{ia1}} } \right)E_{0} \left( {t - t_{{ea}} - t_{{ia2}} - t_{{ia1}} } \right)} \right] - \mu IA_{1} } \hfill \\ {\frac{{dRA_{1} ~}}{{dt}} = \nu _{0} \phi \left( {t_{{ea}} } \right)\phi \left( {t_{{ia2}} } \right)\phi \left( {t_{{ia1}} } \right)\left[ {E_{0} \left( {t - t_{{ea}} - t_{{ia2}} - t_{{ia1}} } \right) - \phi \left( {t_{{ra1}} } \right)E_{0} \left( {t - t_{{ea}} - t_{{ia2}} - t_{{ia1}} - t_{{ra1}} } \right)} \right] - \mu RA_{1} } \hfill \\ \begin{gathered} \frac{{dS_{1} ~}}{{dt}} = \nu _{0} \phi \left( {t_{{ra1}} } \right)\phi \left( {t_{{ea}} } \right)\phi \left( {t_{{ia2}} } \right)\phi \left( {t_{{ia1}} } \right)E_{0} \left( {t - t_{{ea}} - t_{{ia2}} - t_{{ia1}} - t_{{ra1}} } \right) + \nu \phi \left( {t_{{ia3}} } \right)\phi \left( {t_{{ra3}} } \right)E_{1} \left( {t - t_{{ia3}} - t_{{ra3}} } \right) - \left[ {\lambda \left( t \right) + \mu } \right]S_{1} \hfill \\ \frac{{dE_{1} ~}}{{dt}} = \lambda \left( t \right)S_{1} - \left[ {\nu + \mu } \right]E_{1} \hfill \\ \end{gathered} \hfill \\ {\frac{{dIA_{3} ~}}{{dt}} = \nu \left[ {E_{1} \left( t \right) - \phi \left( {t_{{ia3}} } \right)E_{1} \left( {t - t_{{ia3}} } \right)} \right] - \mu IA_{3} } \hfill \\ {\frac{{dRA_{3} ~}}{{dt}} = \nu \phi \left( {t_{{ia3}} } \right)\left[ {E_{1} \left( {t - t_{{ia3}} } \right) - \phi \left( {t_{{ra3}} } \right)E_{1} \left( {t - t_{{ia3}} - t_{{ra3}} } \right)} \right] - \mu RA_{3} } \hfill \\ \end{array} } \right.$$

where $\phi \left( {t_{k} } \right) = \phi_{k} = e^{{ - \mu t_{k} }}$ are survival fractions and $\mu$ is the natural mortality rate; The summation of all equations equals to zero due to the total population $N$ is kept constant. Each differential equation in Eq. (1) describes the variation over time of the number of animals in the considered compartment. The positive and negative terms after the equal sign in each differential equation represent the increase (incoming arrows in Fig. 1) and the decrease (outgoing arrows in Fig. 1) of the number of individuals, respectively, in the considered compartment. To keep the total population constant, all mortality (terms “$- \mu \times compartment$”) is replaced (term “$\mu N$”) by naïve susceptible $S_{0}$. All other terms count for infection and/or transition from one stage to another with transition rates and durations of stays (lag times) in the stages described in Table 1.

The major difference in the dynamics between primo-infected and secondary and later infected manifests itself at two levels:

- Primary infected: the class of exposed animals (infected non-infectious) has two populations: those carrying or not antibodies
- Secondary and subsequent infected: the class of exposed animals (infected non-infectious) has only one population: that which does not carry antibodies. On the other hand, the level of antibodies in the other classes is higher than for the primary infected.

From the combined models, the meaning of variables for the kinetics of infection according to a SEIRS epidemiological model are given by using the following equations (See S1. Methods for the reduced model),

2 $$\left\{ {\begin{array}{*{20}l} {S = S_{0} + S_{1} } \hfill \\ {E = E_{0} + EA_{2} + E_{1} } \hfill \\ {I = IA_{2} + IA_{1} + IA_{3} } \hfill \\ {R = RA_{1} + RA_{3} } \hfill \\ \end{array} } \right.$$

where S, E, I and R are the number of susceptible, exposed, infectious and recovered animals, respectively. The antibody classes are defined from the Fig. 1 as:

3 $$\left\{ {\begin{array}{*{20}l} {{\text{Negative}}\; = \;S_{0} + S_{1} + E_{0} + E_{1} } \hfill \\ {{\text{IgM + IgG}}\;{\text{~}} = \;EA_{2} + IA_{2} } \hfill \\ {{\text{IgG}}\; = \;\left( {EA_{2} + IA_{2} } \right) + \left( {IA_{1} + RA_{1} } \right) + \left( {IA_{3} + RA_{3} } \right)} \hfill \\ {{\text{IgG + }}\;{\text{ = }}\;IA_{3} + RA_{3} } \hfill \\ \end{array} } \right.$$

The basic reproduction number (${R}_{0}$) is an important epidemiologic metric used to describe the transmissibility of infectious disease. It provides the expected number of secondary cases in a naïve population generated by an infectious animal throughout the infectious period. For this system (involving SEIRS model), the basic reproduction number is given by, $R_{0} = \left[ {{\text{prob}}{\text{. to be infected during infectious period}}} \right] \times \left[ {\text{fraction of surviving infectious}} \right] \times \left[ {\text{population size}} \right]$. This reads as:

4 $$R_{0} = \left( {\frac{\beta /N}{{\beta /N + \alpha + \mu }}} \right)\left( {\frac{\alpha }{\alpha + \mu }} \right)N = \left( {\frac{\beta }{{\beta + \left( {\alpha + \mu } \right)N}}} \right)\left( {\frac{\alpha }{\alpha + \mu }} \right)N.$$

Note that when $\beta = 0$, the $R_{0} = 0$, and when $\beta \to \infty$, $R_{0} = \alpha N/\left( {\alpha + \mu } \right)$. In the absence of any information on $\beta$, the $R_{0}$ can be inverted to express the contact-transmission rate $\beta$ as a function of $R_{0}$, population size $N$ and other parameters of the system as:

5 $$\beta = \frac{{R_{0} \left( {\alpha + \mu } \right)^{2} N}}{{\alpha N - R_{0} \left( {\alpha + \mu } \right)}}.$$

From the set of Eq. (1), the meaning and values of parameters for the combined model are provided in Table 1, retrieved from the literature (Table S1). Time lags in Eq. (1) for SEIRS (Eq. S1) and antibody kinetics are constrained as shown in Fig. 1 by the following relations:

6 $$\left\{ {\begin{array}{*{20}l} {t_{e} = \frac{1}{\nu } = \frac{1}{{\nu _{0} }} + \frac{1}{{\nu _{{ea}} }}~~;~t_{{ea}} = \frac{1}{{\nu _{{ea}} }}} \hfill \\ {t_{i} = \frac{1}{\alpha } = t_{{ia2}} + t_{{ia1}} = t_{{ia3}} } \hfill \\ {t_{r} = \frac{1}{\gamma } = t_{{ra1}} = t_{{ra3}} } \hfill \\ {t_{{ea}} + t_{{ia2}} = \frac{1}{{\omega _{2} }}} \hfill \\ {t_{{ia3}} + t_{{ra3}} = \frac{1}{{\omega _{1} }} = \frac{1}{{\omega _{2} }} + t_{{ia1}} + t_{{ra1}} } \hfill \\ \end{array} } \right.$$

To account for the variability and uncertainty in durations $d = t_{ea}$, $t_{i}$, $t_{r}$ and $1/\omega_{2}$ in Table 1, the $d$ were all sampled using a Weibull distribution, $W\left( d \right) = \frac{k}{{\lambda_{d} }}\left( {\frac{d}{{\lambda_{d} }}} \right)^{k - 1} \exp \left[ { - \left( {\frac{d}{{\lambda_{d} }}} \right)^{k} } \right]$, with the shape parameter $k = 2$ and scale parameter, $\lambda_{d} = \frac{2}{\sqrt \pi } \times \left( {\widehat{{t_{ea} }},\widehat{{t_{i} }},\widehat{{t_{r} }},\widehat{{1/\omega_{2} }},} \right)$, where $\hat{ \cdots }$ designates the corresponding mean value given in Table 1. The other parameters were calculated using the relations in Eq. (6).

### Data analysis

The diagnosis of leptospirosis is complicated and depends on the laboratory test. To detect antibodies, there are tests such as microscopic agglutination test (MAT) and immuno-enzymatic assays (ELISA) that are well-known methods. Here, we consider a diagnostic test that would provide one, two or all three of the following outcomes or test results (TR) as shown in Fig. 1: $TR_{1}$—detecting only IgM (and assuming that IgG are also present) and corresponding to the total number of $EA_{2}$ and $IA_{2}$ as $A_{2}$; $TR_{2}$—detecting low levels of IgG corresponding to the number of $IA_{1}$ and $RA_{1}$ as $A_{1}$; and $TR_{3}$—detecting high levels of IgG corresponding to the number of $IA_{3}$ and $RA_{3}$ as $A_{3} .$

**Figure 6:**
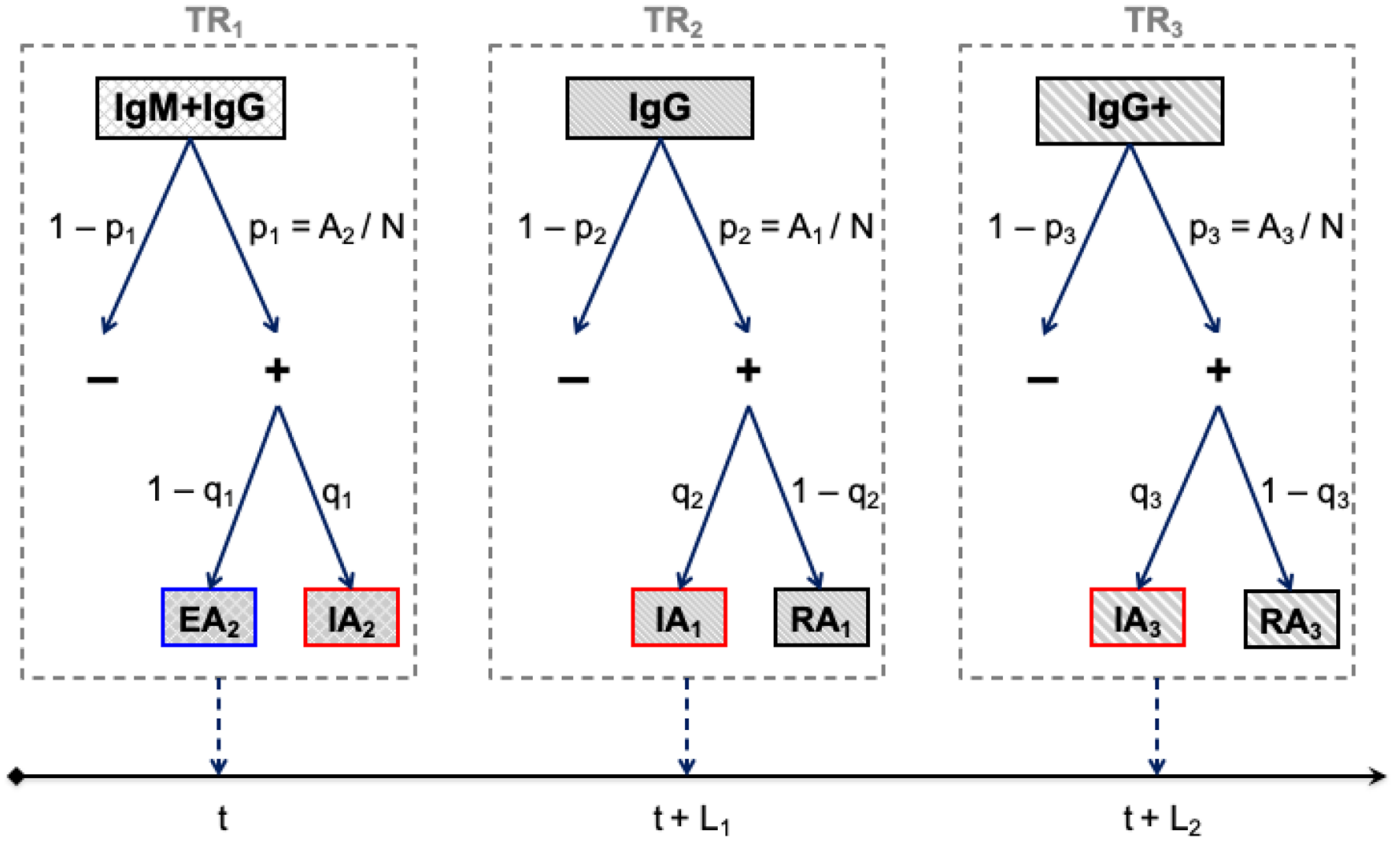
Results of a diagnostic test. TR_i_ stands for test result. The prevalence ${p}_{i}$ corresponds to the true fraction of positive individuals carrying targeted antibodies and ${q}_{i}$ the fraction of them that are infected. ${L}_{1}$ and ${L}_{2}$ represent the lag times between associated antibody states of the population.

As shown in Fig. 6, such a diagnostic test provides information regarding the prevalence ${p}_{i}$ or the fraction of (real or true) positive animals carrying targeted antibodies, the fraction ${q}_{i}$ of positive animals that are infected, and the lag time ($L_{1}$ and $L_{2}$) since infection. To extract that information, the reasoning below goes as follows.

- *Prevalence of antibody positive:* any diagnostic test is characterized by a sensitivity $Se$ and specificity $Sp$. Therefore, the fraction of positive $T_{ + ,i}$ from the diagnostic test result is given by,
  7 $$T_{ + ,i} = Se_{i} \times p_{i} + \left( {1 - Sp_{i} } \right) \times \left( {1 - p_{i} } \right),$$
  where $p_{i}$ is the prevalence or the fraction of real or true positive from test $i$. Now, inverting Eq. (7) provides $p_{i}$ as,
  8 $$p_{i} = \left( {T_{ + ,i} + Sp_{i} - 1} \right)/\left( {Se_{i} + Sp_{i} - 1} \right).$$
  Then, using outputs of simulations, we can use the determined prevalence $p_{i}$ to infer the value of $R_{0}$.
- *Fraction of infected:* using simulation outputs, we can determine the fraction of infected $q_{i}$ corresponding to the determined $R_{0}$.
- *Time lag since infection (for a test performed at time* $t$*)*: $TR_{1}$ identifies freshly infected animals $A_{2}$, and $A_{1}$ and $A_{3}$ positive animals should be observed later at $L_{1}$ and $L_{2}$, respectively. $TR_{2}$ reflects animals $A_{1}$ infected about $L_{1}$ ago that is expected to be $A_{3}$ positive later at $L_{2} - L_{1}$. $TR_{3}$ identifies animals $A_{3}$ infected at least about $L_{2}$ ago.

At the steady state, the expressions of $p_{i}$ are given in Eq. (S5). However, we found that simulations of prevalence of antibody positive can be fitted as well with the power law,

9 $$p_{i} = a_{i} \left( {1 - 1/R_{0} } \right)^{{k_{i} }} ,$$

where $a_{i}$ and $k_{i}$ are given in Table 2.

**Table 2:** The parameters for $p_{i} = a_{i} \left( {1 - 1/R_{0} } \right)^{k}$.

$p_{i}$	$a_{i}$ (95% CI)	$k_{i}$ (95% CI)	$r^{2}$
$p_{1}$	0.0431 (0.0430–0.0432)	0.3530 (0.3442–0.3618)	0.9985
$p_{2}$	0.3626 (0.361–0.3642)	0.3625 (0.3502–0.3748)	0.9973
$p_{3}$	0.5680 (0.5614–0.5746)	1.1000 (1.0580–1.1410)	0.9976

Likewise, the expressions of the fraction of infected ${q}_{i}$ at the steady state are given in Eq. (S6) as,

10 $$\left\{ \begin{gathered} q_{1,s} = \frac{{IA_{2} }}{{IA_{2} + EA_{2} }} = \frac{{\phi_{ea} \left[ {1 - \phi_{ia2} } \right]}}{{1 - \phi_{ea} \phi_{ia2} }} \hfill \\ q_{2,s} = \frac{{IA_{1} }}{{IA_{1} + RA_{1} }} = \frac{{1 - \phi_{ia1} }}{{1 - \phi_{ia1} \phi_{ra1} }} \hfill \\ q_{3,s} = \frac{{IA_{3} }}{{IA_{3} + RA_{3} }} = \frac{{1 - \phi_{ia3} }}{{1 - \phi_{ia3} \phi_{ra3} }} \hfill \\ \end{gathered} \right.$$

Clearly, the $q_{i,s}$ are constant as a function of $R_{0}$.

### Simulations details and Statistical analyses

Stochastic simulations numerically solve Eq. (1) with parameters in Table 1 at daily time step and with initial 10,000 individuals over a 30 year period. Unless stated otherwise, all the parameters are kept the same in all simulations except for the contact-transmission $\beta$ value that is varied via $R_{0}$ using Eq. (5). 1000 simulations were used for statistical analysis. The average numbers of each compartment in Eq. (1) were recorded every 30 days to generate a monthly data series. All simulations were performed using the MATLAB R2016b and statistical analyses were performed using R version 4.0.2.

## Supplementary Information

Supplementary Information.

## Data Availability

The datasets generated during analyzed and/or the current study were made available from the corresponding author based on reasonable requests.
