# Supplementary material for: Modeling of the combined dynamics of leptospirosis transmission and seroconversion in herds
Source: Sci Rep. 2022 Sep 16;12:15620. doi: 10.1038/s41598-022-19833-x (PMC9481562; doi:10.1038/s41598-022-19833-x)
Supplement: Supplementary file 1 — Supplementary Information. [file 41598_2022_19833_MOESM1_ESM.pdf]

Supplementary Information

Modeling of the combined dynamics of leptospirosis transmission and seroconversion in herds

Sudarat Chadsuthi[1], Karine Chalvet-Monfray[2,3], Angeli Kodjo[4], Anuwat Wiratsudakul[5], Dominique J. Bicout[6,7,*]

[1] Department of Physics, Research Center for Academic Excellence in Applied Physics, Faculty of Science, Naresuan University, Phitsanulok 65000, Thailand

[2] Université de Lyon, INRAE, VetAgro Sup, UMR EPIA, 69280 Marcy l'Etoile, France

[3] Université Clermont Auvergne, INRAE, VetAgro Sup, UMR EPIA, 63122 Saint Genès Champanelle, France

[4] USC 1233, Laboratoire des Leptospires, VetAgro Sup, 69280 Marcy l'Etoile, France

[5] Department of Clinical Sciences and Public Health, and the Monitoring and Surveillance Center for Zoonotic Diseases in Wildlife and Exotic Animals, Faculty of Veterinary Science, Mahidol University, Nakhon Pathom 73170, Thailand

[6] Univ. Grenoble Alpes, CNRS, Grenoble INP, VetAgro Sup, TIMC, 38000 Grenoble, France

[7] Laue-Langevin Institute, Theory group, 71 Avenue des Martyrs, 38042, Grenoble, France

S1. Method

Table S1 below lists the references used for parameters in the seroconversion dynamics.

Table S1: The list of references from which parameters used in the antibody dynamics are extracted. “dpi” stands for “days post infection”

Definitions	Time	Method	Leptospira	Animal	Reference
Onset of IgM or IgG	9 dpi	MAT	*Leptospira interrogans* serovar *hardjo*	Cattle	Bercovich et al., 1990
Onset of IgG	27 dpi	ELISA	*Leptospira interrogans* serovar *hardjo*	Cattle	Bercovich et al., 1990
Onset of IgM	7 dpi	ELISA	*Leptospira interrogans* serovars *pomona, hardjo* and *tarassovi*	Cattle	Cousins et al., 1985
Duration of IgM positive	3 - 5 weeks	ELISA	*Leptospira interrogans* serovars *pomona, hardjo* and *tarassovi*	Cattle	Cousins et al., 1985
Duration of IgG positive	12 weeks	ELISA	*Leptospira interrogans* serovars *pomona, hardjo* and *tarassovi*	Cattle	Cousins et al., 1985
Low titer of IgM	8 - 10 weeks pi	ELISA	*Leptospira interrogans* serovar *hardjo*	Cattle	Leonard et al., 1993
Leptospires shedding (urine)	Up to a year	Culture	*Leptospira interrogans* serovar *hardjo*	Cattle	Leonard et al., 1993
Duration of IgM positive	4 weeks pi	ELISA	*Leptospira interrogans* serovar *hardjo*	Cattle	Adler at al., 1982
Positive MAT	1 - 3 weeks earlier pi for IgG ELISA	MAT	*Leptospira interrogans* serovar *hardjo*	Cows	Gerritsen et al., 1993

Adler, B., Cousins, D., Faine, S. & Robertson, G. Bovine IgM and IgM response to Leptospira interrogans serovar hardjo as measured by enzyme immunoassay. *Vet. Microbiol.* **7(6)**, 577-585 (1982).

Bercovich, Z, Taaijke, R. & Bokhout B. Evaluation of an ELISA for the diagnosis of experimentally induced and naturally occurring Leptospira hardjo infections in cattle. *Vet. Microbiol.* **21(3)**, 255-262 (1990).

Cousins, D. V, Robertson, G. M. & Hustas, L. The use of the enzyme-linked immunosorbent assay (ELISA) to detect the IgM and IgG antibody response to Leptospira interrogans serovars hardjo, pomona and tarassovi in cattle. *Vet. Microbiol.* **10**, 439–450 (1985).

Gerritsen, M.J., Koopmans, M.J. & Olyhoek, T. Effect of streptomycin treatment on the shedding of and the serologic responses to Leptospira interrogans serovar hardjo subtype hardjobovis in experimentally infected cows. *Vet. Microbiol.* **38(1-2),** 129-138 (1993).

Leonard, F. C., Quinn, P. J., Ellis, W. A. & O’Farrell, K. Association between cessation of leptospiruria in cattle and urinary antibody levels. *Res. Vet. Sci.* **55**, 195–202 (1993)

The combined kinetics of infection and antibodies dynamics model can reduce to the SEIRS epidemiological model in Eq. S1. The SEIRS dynamic is described by the system of equations as:

$$\begin{cases} \frac{dS}{dt} = \mu N + \nu\phi(t_i)\phi(t_r)E(t - t_i - t_r) - \lambda(t)S(t) - \mu S \\ \frac{dE}{dt} = \lambda(t)S(t) - \nu E - \mu E \\ \frac{dI}{dt} = \nu[E(t) - \phi(t_i)E(t - t_i)] - \mu I \\ \frac{dR}{dt} = \nu\phi(t_i)[E(t - t_i) - \phi(t_r)E(t - t_i - t_r)] - \mu R \end{cases} \quad \text{(S1)}$$

where $t_e = 1/\nu$, t_i and t_r are the mean durations of latent, infection and recovery stages., respectively, and $\phi(t_k) = \phi_k = e^{-\mu t_k}$.

S2. Derivation of expressions at the steady state

At the steady state, all the variables S_0, S_1, E_0, EA_2, E_1, IA_1, IA_2, IA_3, RA_1 and RA_3 become time independent and their time derivatives are equal to zero. Therefore, setting all time derivative in the system of differential equations in Eq.(1) of the main text to zero leads to the following relations:

$$\begin{cases} \mu S_0 = \mu N - (\nu_0 + \mu)E_0 \\ \mu EA_2 = \nu_0 E_0[1 - \phi_{ea}] \\ \mu IA_2 = \nu_0 E_0 \phi_{ea}[1 - \phi_{ia2}] \\ \mu IA_1 = \nu_0 E_0 \phi_{ea}\phi_{ia2}[1 - \phi_{ia1}] \\ \mu RA_1 = \nu_0 E_0 \phi_{ea}\phi_{ia2}\phi_{ia1}[1 - \phi_{ra1}] \\ \mu S_1 = \nu_0 E_0 \phi_{ea}\phi_{ia2}\phi_{ia1}\phi_{ra1} + E_1[\nu\phi_{ia3}\phi_{ra3} - (\nu + \mu)] \\ \mu IA_3 = \nu E_1[1 - \phi_{ia3}] \\ \mu RA_3 = \nu E_1 \phi_{ia3}[1 - \phi_{ra3}] \end{cases} \quad \text{(S2)}$$

with the system of coupled equations,

$$\begin{cases} (\lambda + \mu)(\nu_0 + \mu)E_0 = \lambda\mu N \\ (\lambda + \mu)(\nu + \mu)E_1 = \lambda[\nu_0 E_0 \phi_{ea}\phi_{ia2}\phi_{ia1}\phi_{ra1} + \nu E_1 \phi_{ia3}\phi_{ra3}] \end{cases} \quad \text{(S3)}$$

where $\phi(t_k) = \phi_k = e^{-\mu t_k}$. Eq.(S2) provides the steady state values of S_0, S_1, EA_2, IA_1, IA_2, IA_3, RA_1 and RA_3 as functions of E_0 and E_1 where E_0 and E_1 are obtained by solving the non-linear system of equations in Eq.(S3) with λ, defined before Eq.(1), is given by:

$$\lambda = \left(\frac{\beta}{N}\right)[IA_1 + IA_2 + IA_3] = \left(\frac{\beta}{\mu N}\right)\left[\phi_{ea}(1 - \phi_{ia2}\phi_{ia1})\nu_0 E_0 + (1 - \phi_{ia3})\nu E_1\right] \quad \text{(S4)}$$

There are no straightforward analytical expressions of E_0 and E_1 because of non-linearity of Eq.(S3).

For outcomes or results of the diagnostic test, using the definition of A_1, A_2, and A_3 in Fig. 6 and combining Eq.(S2), we derive the expressions for the prevalence for each diagnostic outcome as,

$$\begin{cases} p_{1,s} = \frac{IA_2+EA_2}{N} = \left(1 - \phi_{ea}\phi_{ia2}\right)\left(\frac{\nu_0}{\mu}\right)\left(\frac{E_0}{N}\right) \\ p_{2,s} = \frac{IA_1+RA_1}{N} = \phi_{ea}\phi_{ia2}\left(1 - \phi_{ia1}\phi_{ra1}\right)\left(\frac{\nu_0}{\mu}\right)\left(\frac{E_0}{N}\right) \\ p_{3,s} = \frac{IA_3+RA_3}{N} = \left(1 - \phi_{ia3}\phi_{ra3}\right)\left(\frac{\nu}{\mu}\right)\left(\frac{E_1}{N}\right) \end{cases} \quad \text{(S5)}$$

where E_0 and E_1 are obtained from the solution of Eq.(S3). As we are missing from analytical expressions for E_0 and E_1, the following two-parameters function, $p_{i,s} = a_i(1 - 1/R_0)^{k_i}$, will be used to fit the outcomes of simulations. However, for non-zero prevalence, analytical expressions for the fractions $q_{i,s}$ of infected antibody positive animals in each diagnostic result are derived from definitions in Fig. 6 and combining Eq.(S2) to give,

$$\begin{cases} q_{1,s} = \frac{IA_2}{IA_2+EA_2} = \frac{\phi_{ea}\left[1-\phi_{ia2}\right]}{1-\phi_{ea}\phi_{ia2}} \\ q_{2,s} = \frac{IA_1}{IA_1+RA_1} = \frac{1-\phi_{ia1}}{1-\phi_{ia1}\phi_{ra1}} \\ q_{3,s} = \frac{IA_3}{IA_3+RA_3} = \frac{1-\phi_{ia3}}{1-\phi_{ia3}\phi_{ra3}} \end{cases} \quad \text{(S6)}$$

S3: The supplementary results

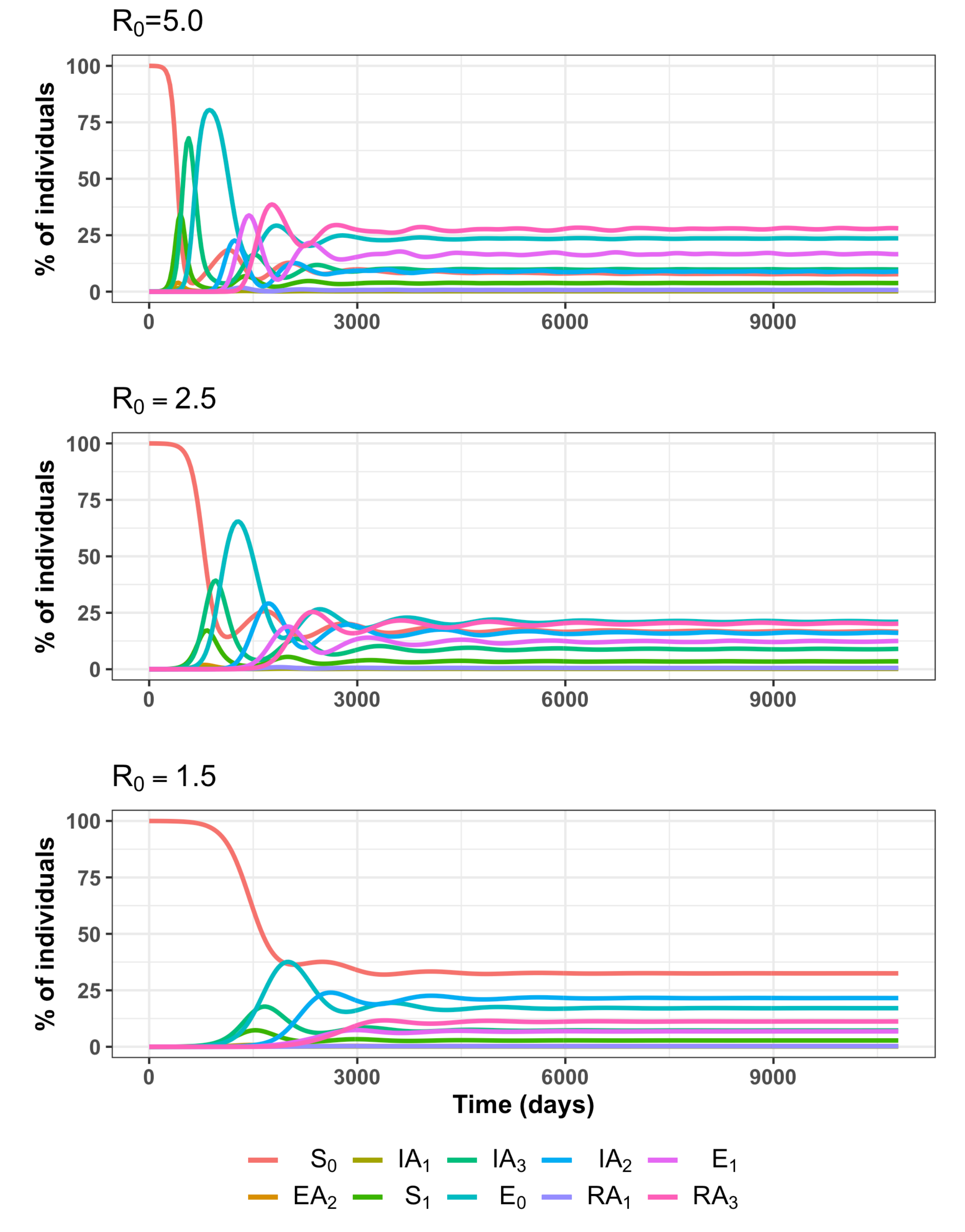

Figure S1. Proportion of individuals as a function of time. Numerical results of full combined model in Eq.(1) of the main text (10 compartments) for R_0= 1.5, 2.5 and 5.0. Proportion (%) = 100 x number of animals / total population; with total population = 10,000.

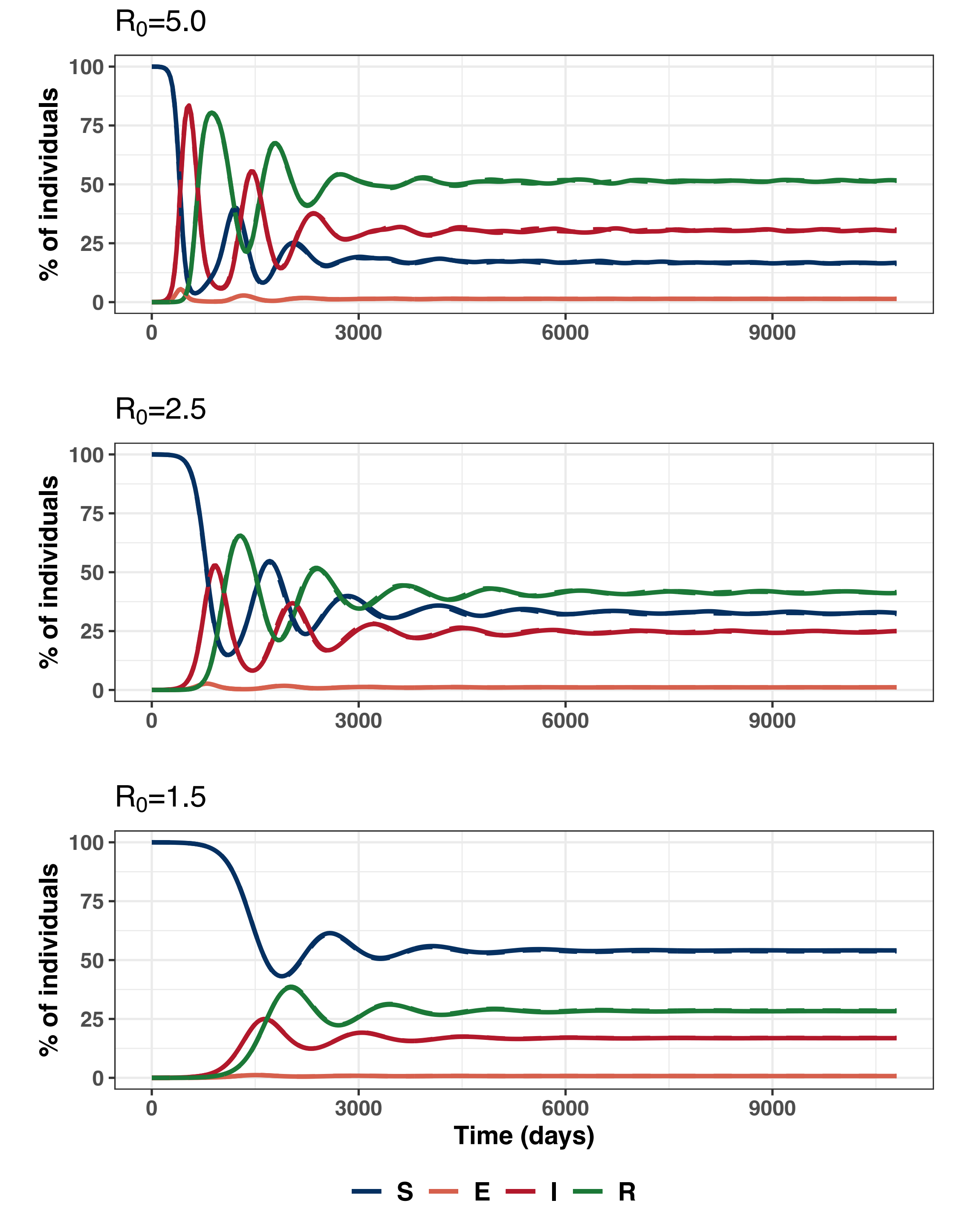

Figure S2. Proportion of individuals as a function of time. The comparison of numerical results between the full combined model in Eq.(1) of the main text (solid lines) with the reduced SEIRS model (dash lines) for R_0= 1.5, 2.5, 5.0 and 7.5. Proportion (%) = 100 x number of animals / total population; with total population = 10,000.

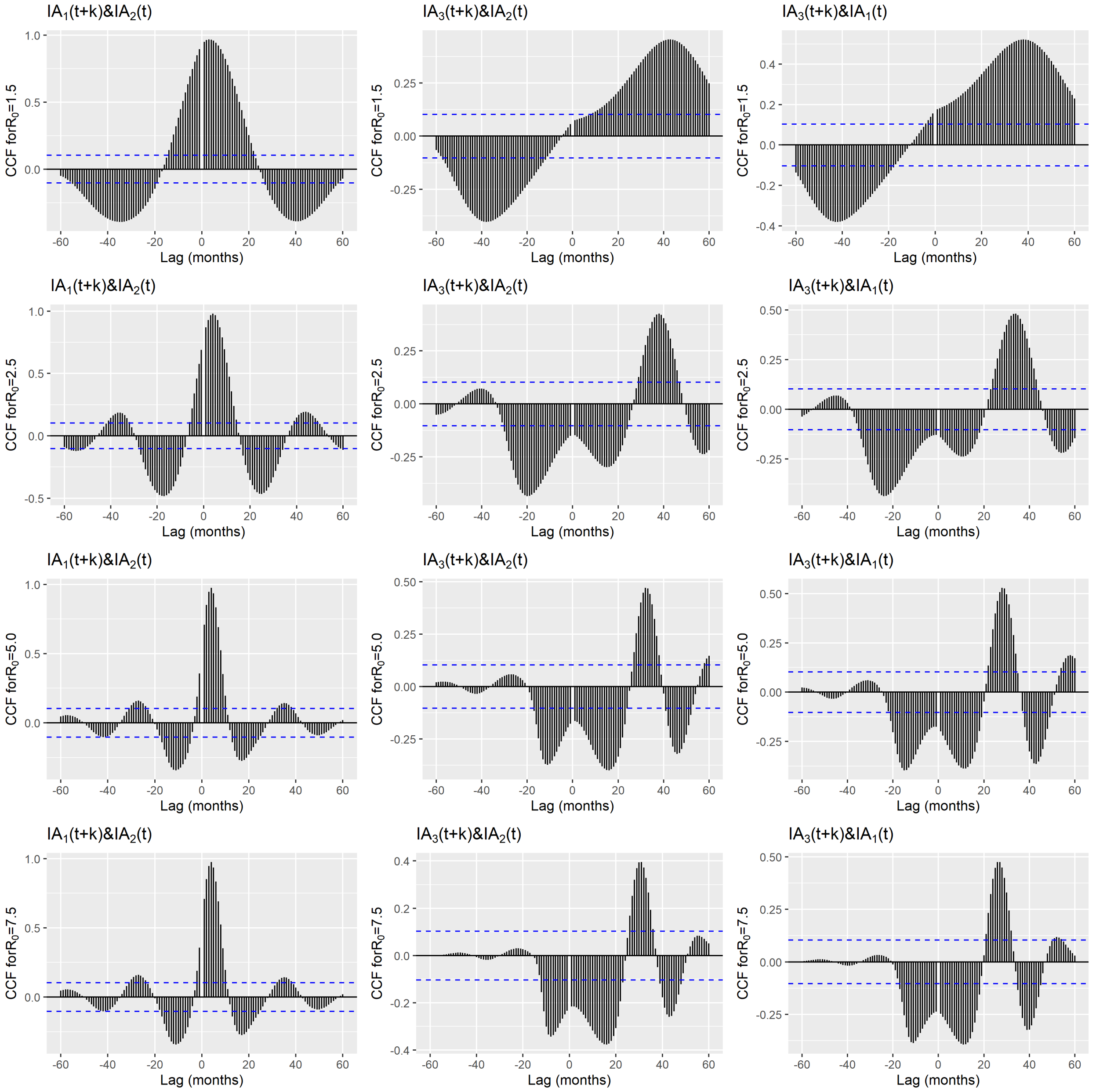

Figure S3. Cross-correlation of infected individuals (IA_2, IA_1 and IA_3) for R_0=1.5, 2.5, 5.0 and 7.5.

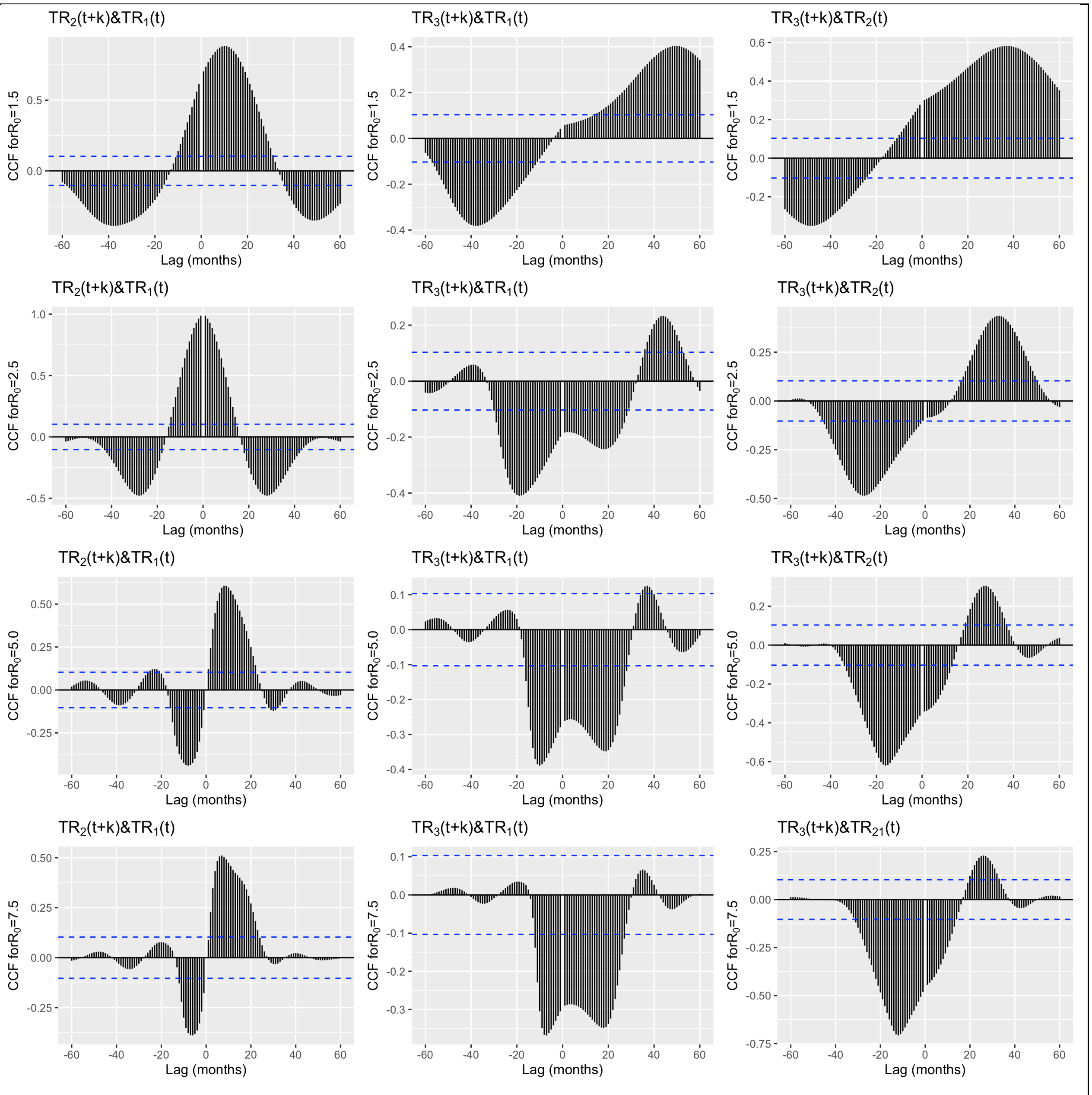

Figure S4. Cross-correlation of diagnostic test results (TR_1, TR_2 and TR_3) for R_0=1.5, 2.5, 5.0 and 7.5.

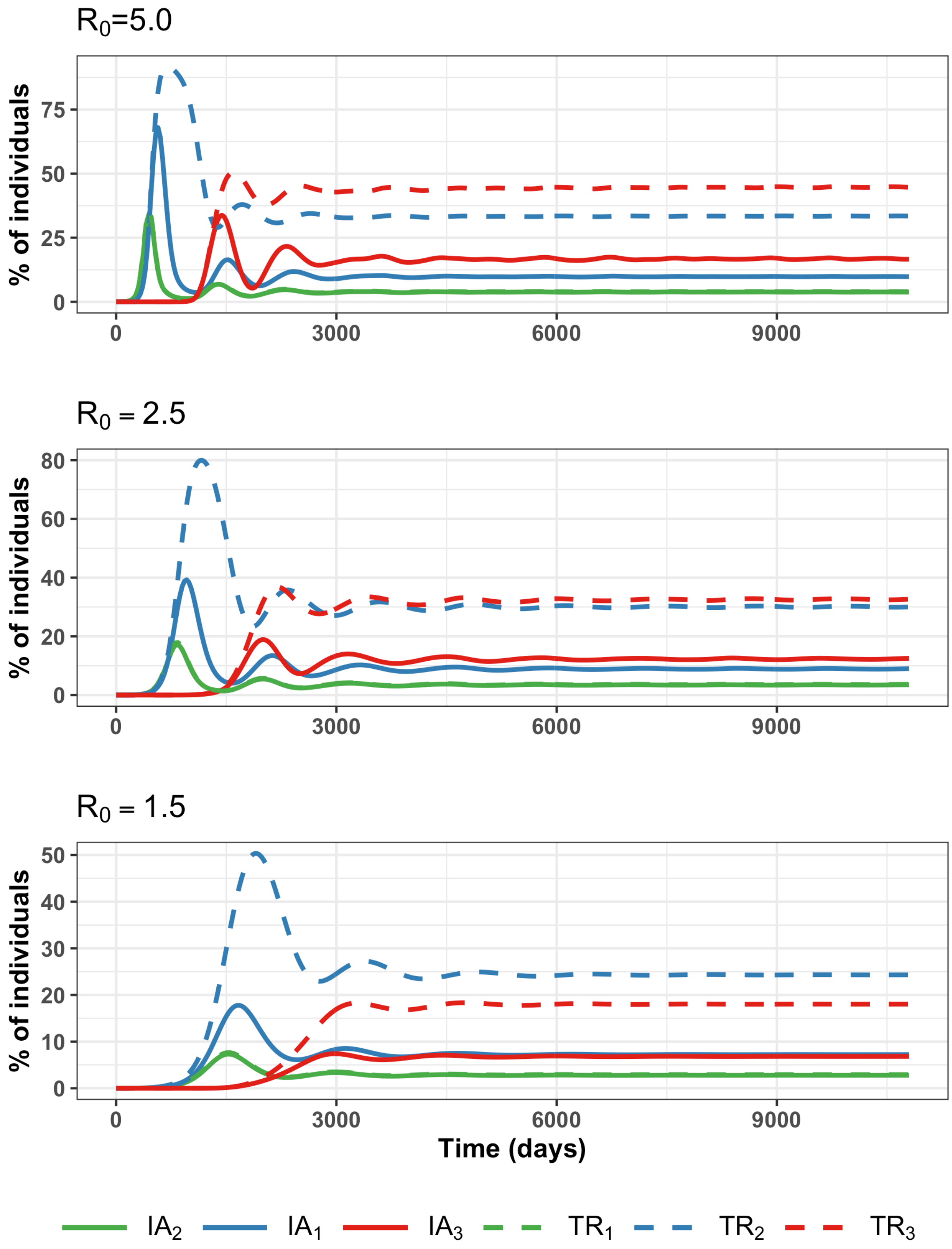

Figure S5. Comparison in the proportion of individuals as a function of time between the number of infectious individuals (solid lines) and the diagnostic test results (dash lines) (IA2 vs TR1, IA1 vs TR2 and IA3 vs TR3) for R_0= 1.5, 2.5, and 5.0. Proportion = 100 x number of animals / total population; with total population = 10,000.

Table S2: The cut-off time (t_c), in days, for each test result TR_i.

R_0	$t_{c,1}$	$t_{c,2}$	$t_{c,3}$
1.5	2310	2760	3900
2.0	1710	2130	3150
2.5	1440	1860	2760
3.0	1290	1680	2490
3.5	1170	1560	2310
4.0	1080	1500	2220
4.5	1020	1440	2100
5.0	960	1380	2040
5.5	900	1350	1980
6.0	870	1320	1920
6.5	810	1320	1860
7.0	780	1290	1890
7.5	750	1290	1770
8.0	720	1260	1740
10.0	630	1230	1680
